# Ethical implications of AI-driven clinical decision support systems on healthcare resource allocation: a qualitative study of healthcare professionals’ perspectives

**DOI:** 10.1186/s12910-024-01151-8

**Published:** 2024-12-21

**Authors:** Cansu Yüksel Elgin, Ceyhun Elgin

**Affiliations:** 1 Department of Ophthalmology, Istanbul University-Cerrahpasa, Istanbul, Turkey; 2 Bogazici University, Istanbul, Turkey; 3 American University in Bulgaria, Blagoevgead, Bulgaria

**Keywords:** Artificial Intelligence, Clinical decision support systems, Healthcare Resource Allocation, Medical Ethics, Qualitative research

## Abstract

**Background:**

Artificial intelligence-driven Clinical Decision Support Systems (AI-CDSS) are increasingly being integrated into healthcare for various purposes, including resource allocation. While these systems promise improved efficiency and decision-making, they also raise significant ethical concerns. This study aims to explore healthcare professionals’ perspectives on the ethical implications of using AI-CDSS for healthcare resource allocation.

**Methods:**

We conducted semi-structured qualitative interviews with 23 healthcare professionals, including physicians, nurses, administrators, and medical ethicists in Turkey. Interviews focused on participants’ views regarding the use of AI-CDSS in resource allocation, potential ethical challenges, and recommendations for responsible implementation. Data were analyzed using thematic analysis.

**Results:**

Participant responses are clustered around five pre-determined thematic areas: (1) balancing efficiency and equity in resource allocation, (2) the importance of transparency and explicability in AI-CDSS, (3) shifting roles and responsibilities in clinical decision-making, (4) ethical considerations in data usage and algorithm development, and (5) balancing cost-effectiveness and patient-centered care. Participants acknowledged the potential of AI-CDSS to optimize resource allocation but expressed concerns about exacerbating healthcare disparities, the need for interpretable AI models, changing professional roles, data privacy, and maintaining individualized care.

**Conclusions:**

The integration of AI-CDSS into healthcare resource allocation presents both opportunities and significant ethical challenges. Our findings underscore the need for robust ethical frameworks, enhanced AI literacy among healthcare professionals, interdisciplinary collaboration, and rigorous monitoring and evaluation processes. Addressing these challenges proactively is crucial for harnessing the potential of AI-CDSS while preserving the fundamental values of equity, transparency, and patient-centered care in healthcare delivery.

**Supplementary Information:**

The online version contains supplementary material available at 10.1186/s12910-024-01151-8.

## Introduction

The integration of Artificial Intelligence-driven Clinical Decision Support Systems (AI-CDSS) into healthcare has sparked a revolution in medical practice, promising enhanced diagnostic accuracy, improved treatment outcomes, and increased operational efficiency. As these systems become more sophisticated and widely adopted, they are increasingly influencing resource allocation decisions and priority setting in healthcare settings. For example, AI-CDSS have been utilized in triage systems to optimize patient prioritization in emergency departments, as demonstrated in a study by Liu et al. [17], where AI models improved efficiency in resource utilization during peak hours. Similarly, Obermeyer et al. [21] highlighted how predictive analytics in AI-CDSS influence the allocation of preventive care resources by identifying high-risk populations. This intersection of AI technology, healthcare economics, and ethical considerations presents a complex landscape that demands careful examination.

AI-CDSS are designed to assist healthcare professionals in making informed decisions by analyzing vast amounts of data and providing evidence-based recommendations. These systems have shown remarkable potential in various medical specialties, including radiology (e.g., detecting anomalies in imaging), oncology (e.g., personalized treatment planning), primary care (e.g., diagnostic decision support), and emergency medicine (e.g., triaging patients in critical conditions. For instance, a study by Ardila et al. [1] demonstrated that an AI model trained to detect lung cancer from CT scans achieved performance on par with, or better than, expert radiologists in a controlled research setting. While this model is not yet integrated into a clinical CDSS, it illustrates the potential impact of AI technologies on healthcare decision-making and resource utilization.

The economic implications of AI-CDSS in healthcare are multi-dimensional. On one hand, these systems promise to reduce healthcare costs by improving diagnostic accuracy, minimizing unnecessary tests and treatments, and optimizing resource allocation. A systematic review by Wolff et al. [32] found that AI-CDSS could lead to substantial cost savings in various healthcare settings. On the other hand, the initial investment required for implementing these systems, along with ongoing maintenance and training costs, presents significant financial challenges for healthcare institutions, particularly in resource-constrained environments.

Ethical considerations in the deployment of AI-CDSS for resource allocation and priority setting are paramount. As these systems increasingly influence decisions about patient care and resource distribution, such as triaging patients in emergency departments or allocating preventive care resources based on predictive analytics [17, 21], questions arise about fairness, equity, and the potential for algorithmic bias. Rajkomar et al. [25] highlighted the risk of AI systems perpetuating or even exacerbating existing healthcare disparities if not carefully designed and implemented. Moreover, the use of AI in healthcare decision-making raises concerns about patient autonomy, informed consent, and the changing dynamics of the doctor-patient relationship.

The ethical framework for integrating AI-CDSS into healthcare economics must also consider the principles of distributive justice and utilitarianism. As healthcare systems globally grapple with limited resources and growing demand, AI-CDSS could play a crucial role in optimizing resource allocation. However, this optimization must be balanced against individual patient rights and the ethical imperative to provide equitable access to healthcare. The seminal work of Beauchamp and Childress [3] provides a foundation for navigating these complex ethical considerations in the context of emerging healthcare technologies.

Despite the growing body of research on AI in healthcare, there remains a significant gap in our understanding of how healthcare professionals perceive and navigate the ethical implications of AI-CDSS in resource allocation and priority setting. While theoretical frameworks for analyzing algorithmic ethics in healthcare have been developed [19], there is limited empirical research on how healthcare professionals interpret and operationalize these ethical considerations in practice. As McDougall [18] argues, the implementation of AI in healthcare requires not just technical competence but also ‘value flexibility’ - the ability to navigate between algorithmic recommendations and human values in clinical decision-making. Moreover, studies such as those by Selamat et al. [28] have explored clinicians’ attitudes towards AI in general medical practice, however, less attention has been paid to the specific intersection of AI, healthcare economics, and ethics from the perspective of frontline healthcare providers.

While research on AI-CDSS in healthcare has grown substantially, studies specifically examining ethical implications in resource allocation contexts remain limited. Existing literature has primarily focused on three areas: technical implementation of AI-CDSS [31], general ethical considerations in AI healthcare applications [6], and broad stakeholder attitudes toward AI in medicine [28]. However, the intersection of these domains—particularly regarding ethical implications of AI-CDSS in resource allocation—remains understudied. The few studies that have examined AI in healthcare resource allocation have largely focused on technical optimization [32] or economic outcomes, rather than ethical implications. While scholars like McDougall [18] have provided theoretical frameworks for considering value conflicts in medical AI, empirical research examining how healthcare professionals navigate these challenges in resource allocation contexts is notably absent. This gap is significant given that resource allocation decisions often involve complex ethical trade-offs between efficiency, equity, and individual patient needs. Furthermore, while studies have examined healthcare professionals’ general attitudes toward AI [28], their perspectives on ethical challenges specific to resource allocation remain unexplored. Resource allocation decisions differ from other clinical applications of AI-CDSS in their distinct ethical implications, often involving competing priorities between system-level efficiency and individual patient care. These unique considerations warrant specific investigation. Additionally, existing research has typically examined clinical decision-making or resource allocation in isolation, rather than investigating the complex interactions between these domains when AI-CDSS is involved. As healthcare systems increasingly deploy AI-CDSS for both clinical and resource allocation decisions, understanding how healthcare professionals perceive and navigate the ethical implications of these intersecting applications becomes increasingly important. The need for research in this area is particularly pressing given the growing adoption of AI-CDSS in healthcare resource allocation decisions. While studies have documented the technical capabilities and potential benefits of these systems [2], there remains limited understanding of how healthcare professionals interpret and address the ethical challenges that arise when AI-CDSS influences resource allocation decisions. This gap in knowledge hampers the development of effective guidelines and frameworks for the ethical implementation of AI-CDSS in resource allocation contexts.

This study aims to address this research gap by exploring healthcare professionals’ perspectives on the ethical implications of AI-CDSS in healthcare resource allocation. Through qualitative interviews with a diverse group of healthcare providers, we seek to uncover the detailed views, concerns, and recommendations of those who are at the forefront of implementing these technologies in clinical practice. By doing so, we hope to contribute to the development of ethical frameworks and guidelines that can inform the responsible integration of AI-CDSS in healthcare financing and resource allocation decisions.

The findings of this study have the potential to inform policy makers, healthcare administrators, and AI developers about the ethical considerations that must be addressed as AI-CDSS become more prevalent in healthcare systems. Moreover, by exploring the perspectives of healthcare professionals, this research can help bridge the gap between theoretical ethical frameworks and the practical realities of clinical decision-making in resource-constrained environments.

As we navigate the complex landscape of AI in healthcare, it is crucial that we continue to critically examine the ethical implications of these technologies, particularly in the context of resource allocation and priority setting. This study aims to contribute to ongoing discussions about the integration of AI-CDSS in healthcare by highlighting the perspectives of healthcare professionals on ethical challenges. While our findings offer valuable insights into stakeholders’ views, we acknowledge that further work is needed to translate these empirical findings into actionable ethical frameworks. Understanding these perspectives is a critical first step, but developing robust ethical guidelines will require interdisciplinary collaboration, synthesis of theoretical and practical insights, and iterative refinement based on real-world implementation.

## Methods

This study employed a qualitative research design to explore healthcare professionals’ perspectives on the ethical implications of AI-driven Clinical Decision Support Systems (AI-CDSS) in healthcare resource allocation. We conducted semi-structured interviews with a diverse group of healthcare providers to gain in-depth insights into their views, concerns, and recommendations regarding the intersection of AI, healthcare economics, and ethics. The interview protocol used for this study can be found among the supplementary materials.

Participant recruitment focused on healthcare professionals with experience or knowledge of AI-CDSS implementation in their practice. We used a purposive sampling strategy to ensure a diverse range of perspectives across different medical specialties, levels of experience, and healthcare settings. Participants were recruited through professional networks, medical associations, and healthcare institutions. The inclusion criteria required participants to be licensed healthcare professionals with at least two years of clinical experience and familiarity with AI-CDSS concepts. The inclusion criteria required participants to be licensed healthcare professionals with at least two years of clinical experience. This was assessed by asking participants to report their professional background and the number of years they had been practicing in their respective fields. In addition, participants were required to have familiarity with AI-CDSS concepts. Familiarity was assessed through a brief pre-interview questionnaire, which asked participants about their experience with AI technologies in healthcare, including any direct or indirect use of AI-CDSS in their clinical practice or decision-making. While there was no strict threshold for the degree of familiarity, participants were required to demonstrate a basic understanding of AI-CDSS systems, such as how these systems support clinical decisions or assist in resource allocation. We aimed for a sample size of 20–25 participants, in line with recommendations for qualitative studies seeking thematic saturation [12]. Since our study employed a deductive approach with predefined thematic areas derived from the interview protocol, thematic saturation was assessed by ensuring that each predetermined theme was fully explored in the interviews. Saturation was reached when no additional insights or information related to these themes were emerging from further interviews, indicating that the sample size was sufficient to cover all aspects of the predefined themes. This process is consistent with Hsieh and Shannon [13]’s approach to thematic analysis, where the analysis is guided by the research questions and pre-determined themes. Both authors reviewed the themes and interview data to confirm that each theme had been sufficiently explored and that no new data was adding to the understanding of the existing themes.

The interview guide was developed based on a comprehensive literature review and in consultation with experts in medical ethics, health economics, and AI in healthcare. The development of our interview protocol was informed by existing theoretical frameworks on algorithmic ethics in healthcare [19] and contemporary debates about value considerations in medical AI implementation [18]. The guide consisted of open-ended questions designed to elicit participants’ views on the ethical considerations of using AI-CDSS for resource allocation and priority setting. Topics covered included perceptions of AI-CDSS impact on healthcare disparities, challenges in balancing cost-effectiveness with patient rights, and views on the ethical framework needed for integrating AI-CDSS in healthcare decision-making. The interview guide was pilot-tested with three healthcare professionals not included in the final sample, and minor adjustments were made based on their feedback to ensure clarity and comprehensiveness. Data collection took place over a period of four months. Due to the geographical dispersion of participants, interviews were conducted via secure video conferencing platforms. Each interview lasted between 45 and 60 min and was audio-recorded with the participant’s consent. The interviewer, trained in qualitative research methods, took field notes during and immediately after each interview to capture non-verbal cues and initial impressions. All interviews were transcribed verbatim by a professional transcription service, with personally identifiable information removed to ensure participant confidentiality.

Given the structured nature of the interview protocol, the study adopted a deductive approach to thematic analysis. While this ensured comprehensive exploration of predefined ethical challenges, it limited the potential for uncovering entirely novel themes. This methodological choice reflects the study’s aim of deepening understanding of known challenges rather than generating new theoretical frameworks. To address this limitation, we focused on eliciting rich, practice-based reflections from participants to enhance the depth of the analysis. Our analysis followed a directed qualitative content analysis approach as described by Hsieh and Shannon [13], which is particularly suitable for studies where existing theory or prior research guides the initial coding framework. This methodology was chosen for its alignment with our deductive approach and structured interview protocol, as it explicitly acknowledges the use of predetermined theoretical frameworks while remaining open to emergent insights. Following Hsieh and Shannon’s [13] guidelines for directed content analysis, our analytical process began with initial coding using predetermined categories derived from existing literature and our interview guide. We then developed detailed operational definitions for each coding category to ensure consistency. These definitions were iteratively refined through discussions between both authors until consensus was reached. The next phase involved detailed review and refinement of coding through regular meetings between authors, where coding decisions were discussed and debated until agreement was achieved. Throughout this process, we identified exemplar quotes and cases that best illustrated each coding category. As recommended by Hsieh and Shannon [13], we paid particular attention to any data that could not be categorized within the initial coding scheme, allowing for the identification of new insights beyond our predetermined framework. Independent verification of coding by both authors served as a final quality check. While we initially considered using Braun and Clarke’s thematic analysis approach (see Braun and Clarke [4, 5], our structured, theory-guided investigation better aligned with Hsieh and Shannon’s directed content analysis methodology. To ensure methodological rigor, we employed strategies outlined by Morse [20] for ensuring trustworthiness in qualitative healthcare research. These included maintaining methodological coherence by aligning our method with research questions, ensuring sampling sufficiency through adequate data saturation, conducting concurrent collection and analysis of data, confirming findings against existing knowledge through theoretical thinking, and moving beyond description to interpretation in our theory development.

To enhance the trustworthiness of our findings, we employed several strategies recommended by Lincoln and Guba [16]. Credibility was ensured through member checking, where a summary of the preliminary findings was sent to participants for verification and feedback. Transferability was addressed by providing thick descriptions of the research context and participant characteristics. Dependability was maintained through an audit trail of the research process, including raw data, analysis notes, and reflexive journals. Confirmability was enhanced with both authors involved in the coding and theme development process. Table C1 in the appendix provides a description of the coding framework.

Ethical considerations were paramount throughout the study. The research protocol was reviewed and approved by the Institutional Review Board of Bogazici University (approval number: 2022-54). Informed consent was obtained from all participants prior to the interviews. Participants were assured of confidentiality and anonymity, and they were informed of their right to withdraw from the study at any time without consequences. Data were stored securely on encrypted devices, and access was limited to the authors only.

To mitigate potential researcher bias, we engaged in reflexive practice throughout the study. The research team consisted of the two authors, who are experienced in medical ethics, health informatics, and qualitative research. Throughout the study, the authors regularly held debriefing sessions to discuss and challenge emerging interpretations, drawing on their complementary expertise. Specifically, one author contributed expertise in medical ethics and the development of the interview guide, while the other author focused on thematic analysis and contextualization within health informatics and qualitative research methodologies. While the primary analysis was conducted by the two authors, informal feedback were sought from external experts during the research process, particularly during the refinement of the thematic coding framework. For example, a discussion with a bioethicist colleague helped challenge initial assumptions about participants’ views on distributive justice, leading to a deeper exploration of equity-related themes. Similarly, feedback from a qualitative research expert informed our decision to revise the coding structure to better differentiate between efficiency and equity concerns. These external contributions are acknowledged below but did not constitute formal authorship roles. We also acknowledged our own positionality and potential biases, particularly regarding our views on AI in healthcare, and actively sought to bracket these during data collection and analysis.

By employing these rigorous qualitative methods, we aimed to generate insights into healthcare professionals’ perspectives on the ethical implications of AI-CDSS in resource allocation. These findings provide a foundation for understanding healthcare professionals’ perspectives on AI-CDSS, which is a crucial element in developing ethical frameworks and guidelines. However, we acknowledge that translating empirical findings into normative frameworks is a complex process that requires additional steps, including philosophical analysis, stakeholder engagement, and integration with existing ethical theories. While this study identifies key themes and concerns, it does not aim to provide a comprehensive or prescriptive ethical framework. Instead, it highlights areas where future interdisciplinary work is needed.

### Methodological reflections

This study employs a qualitative approach to explore healthcare professionals’ perspectives on the ethical implications of AI-CDSS in resource allocation. In conducting this research, we acknowledge that our own professional backgrounds and pre-existing understanding of the topic have likely influenced both the design and interpretation of the study. The first author’s expertise in medical ethics shaped the focus on equity, transparency, and accountability, while the second author’s experience in health informatics guided the exploration of practical challenges in AI implementation.

Recognizing the potential for these perspectives to introduce bias, we adopted several strategies to mitigate their influence. First, the interview guide was developed through a collaborative process that included input from external experts in bioethics and qualitative research, ensuring that it addressed a broad range of relevant issues rather than solely reflecting our own priorities. Second, regular debriefing sessions were held during the data analysis phase to critically examine emerging themes, challenge assumptions, and ensure that the findings accurately reflected the participants’ views. For example, during early coding, our interpretation of participants’ concerns about bias was challenged by the observation that some quotes reflected broader frustrations with healthcare inequities rather than AI-specific issues. This led to a more deeper analysis that distinguished between general equity concerns and those directly linked to AI-CDSS.

Additionally, we recognize the methodological implications of using a deductive approach informed by a structured interview protocol. While this approach allowed us to explore known ethical challenges in depth, it also constrained the emergence of entirely novel themes. We deliberately framed our findings as descriptive insights into stakeholders’ reflections rather than exhaustive accounts of the ethical landscape, acknowledging that they are shaped by the structure and framing of the interviews. This reflective stance aims to enhance the transparency and trustworthiness of our research.

## Results

The results of our qualitative study revealed a complex landscape of perspectives among healthcare professionals regarding the ethical implications of AI-driven Clinical Decision Support Systems (AI-CDSS) in healthcare resource allocation. Through our analysis of the interview data, we identified several key themes that elucidate the views, concerns, and recommendations of our participants.

Our study included 23 healthcare professionals from diverse backgrounds and specialties. The sample comprised 10 physicians, 6 nurses, 4 healthcare administrators, and 3 medical ethicists. The average age of participants was 42 years (range: 28–61), with a mean of 15 years of professional experience (range: 3–35). Participants represented various healthcare settings, including academic medical centers, community hospitals, and private practices, ensuring a broad spectrum of perspectives. Table 1 provides information about participants characteristics.

**Table 1:** Participant characteristics

Characteristic	Number (%)
Profession	
- Physician	10 (43.5%)
- Nurse	6 (26.1%)
- Administrator	4 (17.4%)
- Ethicist	3 (13.0%)
Gender	
- Male	12 (52.2%)
- Female	11 (47.8%)
Age Range	
− 25–34	5 (21.7%)
− 35–44	8 (34.8%)
− 45–54	6 (26.1%)
− 55+	4 (17.4%)
Years of Experience	
− 0–5	3 (13.0%)
− 6–10	5 (21.7%)
− 11–20	9 (39.1%)
− 20+	6 (26.1%)

Participants in this study were healthcare professionals with experience or knowledge of AI-CDSS implementation in their practice. This criterion was crucial for ensuring that participants could provide informed perspectives on the ethical implications of AI-CDSS in healthcare resource allocation. Specifically, participants were selected based on their involvement in clinical decision-making processes where AI-CDSS was integrated. Participants were identified through a combination of methods: (1) contacts with major hospitals in the country that had implemented AI-CDSS systems within the past three years, (2) referrals from a member of the Turkish Medical Informatics Association, and (3) snowball sampling where initial participants recommended colleagues with relevant experience. The recruitment process involved sending formal invitations to potential participants, followed by screening interviews to verify their AI-CDSS experience. Some had directly used AI-CDSS tools, while others had participated in decision-making or oversight roles related to the adoption and deployment of these technologies in healthcare settings. Recruitment was conducted using a purposive sampling strategy, targeting individuals from diverse professional backgrounds (e.g., physicians, nurses, administrators, and medical ethicists) to capture a range of insights. Participants were identified through professional networks, medical associations, and institutional affiliations known to be early adopters of AI technologies. Invitations were sent to prospective participants via email, detailing the study objectives and inclusion criteria. To ensure credibility, we selected participants with at least two years of clinical experience and prior exposure to discussions or decisions regarding AI-CDSS. While participants were not required to have experience specifically with AI-CDSS for resource allocation, all were familiar with the broader use of AI-CDSS in clinical settings. Among those interviewed, several described scenarios where AI-CDSS influenced resource distribution indirectly—such as triaging patients or prioritizing diagnostic interventions—providing valuable reflections on resource allocation ethics.

Had we selected participants with less direct knowledge or practical experience with AI-CDSS, the findings might have emphasized general attitudes toward AI rather than nuanced ethical reflections grounded in clinical practice. For instance, individuals with limited familiarity may have raised broader concerns about AI technology or speculative scenarios rather than focusing on the intersection of AI-CDSS and resource allocation. Thus, our sampling approach was designed to elicit rich, experience-based insights while acknowledging that future studies might benefit from contrasting these perspectives with those of less experienced or skeptical stakeholders.

Participants’ responses clustered around five predetermined thematic areas, reflect the key ethical challenges identified in previous literature on AI-CDSS implementation. Their reflections provided valuable insights into how these challenges manifest in daily clinical practice. We should also mention that all quotes presented in this paper have been edited for clarity and readability, ensuring they are concise and accessible to readers while retaining the original meaning. Minor adjustments, such as the removal of filler words or grammatical corrections, were made to improve flow and coherence without altering the content or context of participants’ statements. To maintain transparency, we acknowledge this editing process and provide some unedited, verbatim quotes in the appendix to illustrate participants’ real-time struggles in articulating their reflections and grappling with the ethical dilemmas posed by AI-CDSS.

### Theme 1: balancing efficiency and equity in resource allocation

A predominant theme that emerged from our analysis was the tension between the potential for AI-CDSS to improve healthcare efficiency and the concern for maintaining equitable access to care. Many participants acknowledged the potential of AI-CDSS to optimize resource allocation through data-driven decision-making. For instance, a hospital administrator (Participant 7) stated, “AI systems can process vast amounts of data to identify areas where resources are being underutilized or overutilized, potentially leading to more efficient allocation.” This perspective was particularly common among participants in administrative roles, who frequently highlighted the system’s ability to identify inefficiencies in resource distribution.

However, this optimism was consistently tempered by concerns about the potential for AI-CDSS to exacerbate existing healthcare disparities. A medical ethicist (Participant 18) cautioned, “If we’re not careful, these systems could inadvertently prioritize resources towards populations that are already well-served, further marginalizing vulnerable groups.” This concern was shared across different professional roles, with participants expressing particular worry about automated decision-making potentially disadvantaging certain patient populations.

The need for careful design and implementation of AI-CDSS emerged as a crucial subtheme, with participants emphasizing that efficiency gains should not compromise equity. A primary care physician (Participant 3) suggested, “We need to build safeguards into these systems to actively counteract existing biases and prioritize equitable access to care.” Several participants described developing their own informal protocols to review AI recommendations, particularly for cases involving traditionally underserved populations.

The tension between efficiency and equity manifested differently across various healthcare settings. Participants from resource-constrained settings, such as those from small-city hospitals, consistently prioritized equity concerns over efficiency gains. For example, Participant 15, a critical care nurse, remarked: “In settings like ours, efficiency is meaningless unless equity is addressed. AI has the potential to widen the gap, so we consciously adjust how we use it to serve our most vulnerable patients first.” This perspective was echoed by other participants working in similar settings, who described developing specific strategies to ensure AI recommendations didn’t disadvantage their vulnerable patient populations.

Many participants also noted the practical challenges of balancing these competing priorities in daily practice. They described various informal approaches to mediating between AI recommendations and equity considerations, such as additional review processes for certain patient groups, regular team discussions about AI recommendations, and maintaining manual oversight of resource allocation decisions. These practical strategies revealed how healthcare professionals actively work to maintain equity while leveraging the efficiency benefits of AI-CDSS.

### Theme 2: transparency and explicability of AI-CDSS

Another significant theme that emerged was the importance of transparency and explicability in AI-CDSS used for resource allocation decisions. Participants consistently expressed the need to understand how these systems arrive at their recommendations, particularly when they influence decisions about patient care and resource distribution. This concern was especially pronounced among clinicians who regularly needed to communicate AI-assisted decisions to patients and their families.

A neurologist (Participant 12) emphasized, “If I’m going to rely on an AI system to help me make decisions about resource allocation, I need to be able to understand and explain its reasoning to my patients and colleagues.” This sentiment was echoed across different specialties, with many participants describing specific instances where they struggled to explain AI-generated recommendations to stakeholders.

Several participants raised concerns about the “black box” nature of some AI algorithms and its implications for ethical decision-making. An oncologist (Participant 9) noted, “There’s a risk of deferring too much to these systems without truly understanding their limitations or potential biases.” This concern was particularly acute in cases involving complex resource allocation decisions, where participants reported feeling uncomfortable making decisions they couldn’t fully explain or justify.

To address these transparency challenges, participants described developing various informal and formal strategies. These included creating simplified explanation frameworks for patients, maintaining detailed records of override decisions, and establishing peer review processes for AI recommendations. A healthcare administrator (Participant 20) proposed, “We need to develop a culture of ‘AI literacy’ among healthcare providers, where understanding and critically evaluating these systems becomes a core competency.” Several institutions represented in our study had already begun implementing regular training sessions and establishing guidelines for AI system use.

The need for transparency varied across different contexts and decision types. Participants reported that for routine resource allocation decisions, such as scheduling and basic inventory management, they were generally comfortable with less detailed explanations of AI decision-making. However, for decisions affecting patient care directly or involving significant resource trade-offs, they expressed a strong need for detailed understanding of the AI’s reasoning process. Many participants described developing their own methods for verifying and validating AI recommendations in these high-stakes situations.

Participants also highlighted the practical challenges of maintaining transparency in time-sensitive situations. Several described developing quick reference guides and decision trees to help them rapidly assess AI recommendations while maintaining a basic understanding of the system’s reasoning. These practical solutions revealed how healthcare professionals actively work to balance the need for efficiency with the imperative for transparent and explicable decision-making.

### Theme 3: shifting roles and responsibilities in clinical decision-making

The integration of AI-CDSS into resource allocation processes raised significant questions among participants about the changing nature of clinical decision-making and professional responsibility. Participants across all professional roles expressed complex and often conflicting views about how AI systems were reshaping their professional responsibilities and decision-making autonomy.

A critical care nurse (Participant 15) reflected, “While these systems can provide valuable insights, we can’t lose sight of the importance of human empathy and contextual understanding in healthcare decisions.” This sentiment was particularly strong among frontline healthcare providers, who frequently described situations where they felt the need to balance algorithmic recommendations against their clinical experience and understanding of patient-specific contexts.

Questions of accountability emerged as a central concern when discussing AI-CDSS involvement in resource allocation decisions. An emergency medicine physician (Participant 5) pondered, “If a decision informed by an AI system leads to a negative outcome, who bears the responsibility - the clinician, the hospital, or the system developers?” This uncertainty about accountability was especially pronounced in cases involving complex resource allocation decisions, where multiple stakeholders and competing priorities were involved.

Many participants described developing informal practices to maintain their professional autonomy while utilizing AI recommendations. These included maintaining detailed documentation of their reasoning when overriding AI suggestions, conducting regular team discussions about AI-assisted decisions, and establishing clear protocols for when human judgment should take precedence. A medical ethicist (Participant 22) suggested, “We need to develop a framework that clearly delineates the role of AI as a decision support tool, not a replacement for clinical expertise.”

The shifting nature of professional roles emerged as a particular concern among more experienced healthcare providers. Several participants with over 15 years of clinical experience described feeling challenged by the need to integrate AI recommendations into their established decision-making processes. For instance, an experienced surgeon (Participant 17) noted, “After twenty years of making these decisions based on clinical judgment, it’s not easy to suddenly start sharing that responsibility with an algorithm. We need time to adjust our professional identity to this new reality.”

The discomfort with shifting accountability was particularly evident in emergency and critical care settings. Another emergency physician (Participant 5) questioned: “If a resource allocation decision guided by AI turns out wrong, am I still held responsible? Or does the blame fall on the AI developers?” This concern was echoed across different specialties, with participants consistently expressing the need for clearer institutional guidelines about decision-making authority and professional liability.

Participants also described various strategies they had developed to maintain professional control while leveraging AI capabilities. These included creating decision checkpoints where AI recommendations would be reviewed by senior staff, establishing regular forums for discussing challenging cases, and developing departmental guidelines for AI system use. Several departments had begun implementing formal protocols to clarify the hierarchy of decision-making authority when using AI-CDSS.

#### Theme 4: ethical considerations in data usage and algorithm development

Participants expressed significant concerns about the ethical implications of data usage and algorithm development in AI-CDSS for resource allocation. Issues of patient privacy, consent, and data ownership emerged as primary concerns across all professional groups, with particular emphasis on the complexity of these issues in resource allocation contexts.

A primary care physician (Participant 1) voiced fundamental concerns about patient consent: “Are patients fully aware of how their data might be used in these systems, especially when it comes to influencing resource allocation decisions?” This concern was echoed by several other participants who described specific challenges in explaining to patients how their data might influence future resource allocation decisions. Some participants shared experiences of patients expressing discomfort when learning their data could affect not only their own care but also broader resource distribution decisions.

The need for diverse and representative data sets in AI-CDSS development emerged as another crucial concern. A healthcare administrator (Participant 16) noted, “If these systems are trained on data that doesn’t adequately represent our diverse patient population, we risk perpetuating or even amplifying existing health disparities.” This concern was particularly pronounced among participants working in diverse urban settings, who provided specific examples of how AI recommendations sometimes failed to account for cultural, socioeconomic, and demographic factors specific to their patient populations.

Several participants described developing their own informal monitoring systems to track potential biases in AI recommendations. For instance, a department head (Participant 11) shared: “We’ve started keeping track of cases where the AI recommendations seem misaligned with our patient population’s needs. It’s helped us identify patterns we might have missed otherwise.” These informal monitoring practices varied across institutions but generally included regular review meetings, documentation of override decisions, and tracking of outcomes in different patient subgroups.

The importance of ongoing monitoring and evaluation of AI-CDSS emerged as a key subtheme. A nurse practitioner (Participant 8) suggested, “We need robust mechanisms for continuous assessment of these systems’ impact on resource allocation and patient outcomes.” Participants described various approaches their institutions had implemented or were planning to implement, ranging from monthly audit meetings to detailed tracking systems for AI-assisted decisions.

Participants also raised concerns about data security and privacy in the context of resource allocation decisions. Several described struggling with the balance between gathering comprehensive data for improved decision-making and maintaining patient privacy. A privacy officer (Participant 19) noted: “Every additional piece of data we collect potentially improves the AI’s recommendations, but also increases our privacy obligations and risks. It’s a constant balancing act.”

Many participants emphasized the need for greater transparency in how patient data influences resource allocation algorithms. They described challenges in explaining to patients the relationship between data sharing and resource allocation decisions, with some reporting that patients became more hesitant to share data when they understood its broader implications for resource distribution.

### Theme 5: balancing cost-effectiveness and patient-centered care

The final major theme that emerged from our analysis was the challenge of balancing cost-effectiveness considerations with the principles of patient-centered care when using AI-CDSS for resource allocation. Participants across different roles and specialties described complex tensions between leveraging AI for cost optimization while maintaining personalized, compassionate care delivery.

An oncologist (Participant 14) reflected, “While AI can help us identify the most cost-effective treatments, we must ensure that these recommendations don’t override individual patient preferences and values.” This sentiment was particularly strong among specialists dealing with complex or chronic conditions, where treatment decisions often involved numerous personal and contextual factors that participants felt weren’t adequately captured by AI systems.

The potential for AI-CDSS to affect the balance between financial considerations and patient care emerged as a significant concern. A medical ethicist (Participant 23) cautioned, “There’s a risk that these systems could be used to justify rationing of care under the guise of ‘optimization,’ particularly in resource-constrained settings.” This concern was especially pronounced among participants working in public healthcare facilities and other resource-limited environments, where financial pressures were already significant.

Participants described developing various strategies to maintain patient-centered care while using AI-CDSS. A family physician (Participant 11) suggested, “We need to design these systems to support, not replace, the human elements of care - empathy, communication, and shared decision-making.” Several participants shared specific examples of how they integrated AI recommendations into patient consultations while maintaining focus on individual patient needs and preferences.

The practical implementation of these principles varied across different healthcare settings. A primary care physician (Participant 3) described implementing additional checks to mitigate potential biases: “When the AI system flagged certain patients for resource allocation, I always cross-referenced with non-AI data to ensure fairness, especially in underserved populations.” This approach was echoed by other participants who had developed similar verification processes.

Organizational efforts to address these challenges were also highlighted. A hospital administrator (Participant 7) detailed their institution’s approach: “We organized workshops for our team to understand the algorithms, which helped reduce reliance on the AI as a ‘black box’ and encouraged critical engagement.” Several participants described similar initiatives at their institutions, ranging from regular team discussions about AI recommendations to formal protocols for balancing cost-effectiveness with patient needs.

Participants also emphasized the importance of maintaining flexibility in AI-assisted resource allocation decisions. Many described situations where they had to override cost-effectiveness recommendations to accommodate specific patient circumstances. A nurse manager (Participant 10) shared: “Sometimes the AI suggests the most cost-effective approach, but we know from experience that it won’t work for a particular patient’s situation. We’ve learned to trust our clinical judgment in these cases.”

The challenge of communicating cost-effectiveness decisions to patients emerged as a significant subtheme. Participants described developing various approaches to explain resource allocation decisions while maintaining trust and empathy. A palliative care specialist (Participant 25) noted: “It’s one thing to have an AI system tell you what’s cost-effective, but it’s another thing entirely to have that difficult conversation with a patient or their family. We need to maintain the human touch in these discussions.”

Table D1 in the appendix provide some additional illustrative quotes for different themes presented above. Moreover, Table E1 compares some original quotes agains the edited ones.

In conclusion, our analysis highlights healthcare professionals’ reflections on a structured set of ethical challenges concerning AI-CDSS in healthcare resource allocation, as outlined in the interview protocol. While participants shared diverse viewpoints, these were largely shaped by the predefined themes of the interview guide. This reflects a deductive approach, focusing on eliciting detailed insights into known ethical issues in the context of participants’ professional experiences.

## Discussion

This study provides essential insights into how healthcare professionals interpret and navigate ethical challenges in the implementation of AI-CDSS. Our findings validate existing theoretical frameworks while adding practical dimensions to known challenges, shedding light on strategies used by healthcare professionals to address these issues in real-world contexts.

The recognition by participants of AI-CDSS’s potential to optimize resource allocation aligns with Topol’s [30] analysis of AI’s capacity to enhance healthcare efficiency. Simultaneously, participants expressed concerns about equity, reflecting challenges identified in the literature, such as Obermeyer et al.‘s [21] demonstration of racial biases in AI algorithms. This highlights a persistent tension between efficiency and fairness in AI-driven healthcare. The strategies proposed by participants to counteract biases, such as more equitable dataset representation and regular auditing, align with frameworks proposed by Rajkomar et al. [25] to ensure fairness in machine learning applications in healthcare.

The emphasis placed on transparency and explicability by our participants mirrors the increasing importance of “explainable AI” in medicine. Their insistence on understanding AI system recommendations resonates with Holzinger et al.‘s [14] call for interpretable AI models. Concerns about the “black box” nature of AI systems, as described by Char et al. [6], persist as a significant barrier to trust and adoption. Participants emphasized the need for more interpretable AI models, echoing Rudin’s [27] suggestions, alongside regular audits of outputs as recommended by Gianfrancesco et al. [11]. Moreover, participants highlighted the need for “AI literacy” among healthcare providers, aligning with Kolachalama and Garg’s () recommendation for integrating AI education into medical training to enhance clinicians’ ability to critically evaluate AI outputs.

The findings also illuminate how AI is reshaping clinical decision-making roles. While participants acknowledged the potential benefits of AI-assisted decision-making, they emphasized the importance of maintaining human judgment and empathy. This aligns with Sujan et al.‘s [29] work on the necessity of human agency in AI-supported systems. The concerns raised regarding accountability, particularly in AI-assisted resource allocation decisions, echo broader debates in the literature, such as Price et al.’s [23] analysis of medical malpractice and liability issues. To address these concerns, participants suggested clearer guidelines and frameworks to preserve professional autonomy while integrating AI-CDSS into workflows. Char et al.‘s [6] recommendations for maintaining human judgment in AI-supported decision-making offer a valuable foundation for this endeavor.

The ethical considerations surrounding data usage and consent reflect ongoing challenges highlighted by Cohen et al. [8]. Participants stressed the importance of using diverse and representative datasets to mitigate biases, an issue also raised by Chen et al. [7]. This underscores the need for initiatives like those proposed by Zou and Schiebinger [33] to improve data diversity and fairness. Additionally, concerns about patient privacy and consent point to a broader need for updated frameworks tailored to the ethical complexities of AI in healthcare.

Balancing cost-effectiveness with patient-centered care emerged as another prominent theme. Participants worried that AI-driven efficiency gains might compromise individualized, compassionate care, a concern echoed by [9] in their work on shared decision-making. This highlights the importance of integrating AI-CDSS in ways that enhance rather than diminish the human elements of care, as Reddy et al. [26] suggest. Furthermore, participants identified tensions between financial imperatives and ethical obligations, echoing Persad et al.’s [10] analysis of resource allocation ethics. Ensuring that AI-CDSS supports equitable and compassionate care requires ethical guidelines that balance population-level efficiency with individual patient needs.

Our analysis revealed important patterns in how different stakeholders approached ethical challenges in AI-CDSS implementation. While concerns about equity and efficiency were widespread, their manifestation varied by role and setting. Administrators, exemplified by Participant 7’s focus on identifying resource inefficiencies, tended to view AI-CDSS through the lens of system-level optimization. In contrast, frontline providers in resource-constrained settings, like Participant 15, emphasized protecting vulnerable populations over efficiency gains. This distinction was particularly evident in smaller hospitals and resource-limited environments, where healthcare providers developed specific strategies to ensure AI recommendations didn’t disadvantage vulnerable patients.

The experience level of practitioners also emerged as a crucial factor influencing perspectives on AI-CDSS integration. More experienced clinicians, such as the surgeon (Participant 17) with twenty years of experience, reported greater challenges in incorporating AI recommendations into their established decision-making processes. This suggests that implementation strategies may need to be tailored not only to professional roles but also to practitioners’ experience levels.

Practice setting emerged as another important factor shaping stakeholder perspectives. Providers in urban, diverse settings expressed particular concern about AI systems’ ability to account for varied patient populations, while those in resource-constrained environments focused more on ensuring equitable access to care. These context-specific variations in stakeholder priorities suggest that successful implementation of AI-CDSS requires careful attention to local healthcare environments and resources.

The findings of our study also provide insights into how healthcare professionals interpret and navigate previously identified ethical challenges in AI-CDSS implementation. Rather than discovering novel ethical concerns, our findings illuminate the practical manifestations of known challenges and the strategies healthcare professionals employ to address them. This practical perspective adds valuable context to existing theoretical frameworks for ethical AI implementation in healthcare.

One of the most prominent themes that emerged from our analysis was the tension between the potential for AI-CDSS to improve healthcare efficiency and the concern for maintaining equitable access to care. This dichotomy reflects a broader debate in the field of health economics and ethics regarding the balance between utilitarian and egalitarian approaches to resource allocation [22]. Our participants’ recognition of AI-CDSS’s potential to optimize resource allocation aligns with the growing body of evidence supporting the efficiency gains of these systems. For instance, Beam and Kohane [2] have demonstrated how AI can improve diagnostic accuracy and reduce unnecessary testing, potentially leading to more efficient use of healthcare resources.

However, the concerns raised by our participants about the potential for AI-CDSS to exacerbate existing healthcare disparities are well-founded and echo similar worries expressed in the literature. The work of Obermeyer et al. [21] on racial bias in healthcare algorithms serves as a stark reminder of the potential for AI systems to perpetuate or even amplify existing inequities. This underscores the critical importance of careful design and implementation of AI-CDSS, with a focus on actively counteracting biases and prioritizing equitable access to care. The framework proposed by Rajkomar et al. [25] for ensuring fairness in machine learning applications in healthcare provides a valuable starting point for addressing these concerns.

The emphasis placed by our participants on the need for transparency and explicability in AI-CDSS used for resource allocation decisions reflects a growing recognition of the importance of “explainable AI” in healthcare. This aligns with the work of Holzinger et al. [14], who argue that the ability to interpret and explain AI decisions is crucial for their ethical and practical implementation in medical settings. The concerns raised about the “black box” nature of some AI algorithms echo the challenges identified by Char et al. [6] regarding the ethical implications of opaque decision-making processes in clinical care.

Addressing these concerns will require a multi-dimesional approach. The development of more interpretable AI models, as suggested by Rudin [27], could help improve transparency. Regular audits of AI-CDSS outputs, as proposed by Gianfrancesco et al. [11], could help detect and mitigate biases. Furthermore, the call from our participants for comprehensive training to develop “AI literacy” among healthcare providers aligns with recommendations from Kolachalama and Garg [15] for integrating AI education into medical curricula.

The theme of shifting roles and responsibilities in clinical decision-making raises important questions about the nature of medical expertise and professional autonomy in the age of AI. The tension expressed by our participants between the potential benefits of AI-assisted decision-making and the importance of maintaining human judgment and empathy reflects broader debates in the field. Sujan et al. [29] have emphasized the need to maintain human agency in AI-supported healthcare systems, arguing that the contextual understanding and ethical reasoning provided by human clinicians remain essential.

The questions of accountability raised by our participants when AI-CDSS are involved in resource allocation decisions touch on complex legal and ethical issues. As Price [24] has discussed, the integration of AI into clinical practice challenges traditional notions of medical malpractice and liability. Developing clear guidelines and protocols for integrating AI-CDSS into clinical workflows while preserving professional autonomy and judgment will be crucial. The framework proposed by Char et al. [6] for maintaining the centrality of human judgment in AI-assisted clinical decision-making provides a valuable starting point for addressing these challenges.

The ethical considerations surrounding data usage and algorithm development in AI-CDSS, as highlighted by our participants, reflect growing concerns in the field of health informatics. The issues of patient privacy, consent, and data ownership raised in our study align with the ethical challenges of big data analytics in healthcare discussed by Cohen et al. [8]. Addressing these concerns will require careful consideration of existing ethical frameworks and potentially the development of new ones tailored to the unique challenges posed by AI in healthcare.

The need for diverse and representative data sets in the development of AI-CDSS, emphasized by our participants, is crucial for ensuring fair and unbiased outcomes. This aligns with the work of Chen et al. [7]. Initiatives to improve data diversity and representation, such as those proposed by Zou and Schiebinger [33], will be essential for developing AI-CDSS that can effectively serve diverse patient populations.

The challenge of balancing cost-effectiveness considerations with the principles of patient-centered care when using AI-CDSS for resource allocation reflects a fundamental tension in healthcare delivery. The concern expressed by our participants about maintaining individualized, compassionate care in the face of AI-driven efficiency aligns with the principles of shared decision-making in healthcare, as discussed by Elwyn et al. [9]. Integrating AI-CDSS into clinical practice in a way that supports rather than replaces the human elements of care will be crucial for maintaining a patient-centered approach.

The potential for AI-CDSS to exacerbate existing tensions between financial considerations and ethical obligations in healthcare, as noted by our participants, touches on longstanding debates in health policy and ethics. The work of Persad et al. [10] on fair allocation of scarce medical resources provides a valuable framework for considering these issues. As AI-CDSS become more prevalent in resource allocation decisions, it will be essential to develop ethical guidelines that balance population-level outcomes with individual patient needs and preferences.

Our participants described several concrete elements of what constitutes a holistic approach to resource allocation. This approach, as evidenced in our findings, involves multiple integrated components: First, the implementation of multi-level review processes, as illustrated by the primary care physician (Participant 3) who described cross-referencing AI recommendations with non-AI data to ensure fairness for underserved populations. Second, the development of formal and informal strategies to maintain flexibility in decision-making, exemplified by the nurse manager (Participant 10) who described overriding cost-effectiveness recommendations based on patient-specific circumstances. Third, the establishment of regular team discussions and forums for evaluating AI recommendations, as highlighted by several institutions’ practices. Fourth, the integration of ongoing monitoring systems, as described by the department head (Participant 11) who tracked cases where AI recommendations didn’t align with patient population needs. This multi-faceted approach differs from existing frameworks by emphasizing active, continuous engagement with AI recommendations rather than passive implementation, and by incorporating both formal institutional protocols and informal professional judgment. The work of Reddy et al. [26] on artificial intelligence-enabled care delivery provides valuable insights into how such integrated approaches can enhance rather than diminish the human aspects of care.

Our findings have several important implications for policy and practice. First, they underscore the need for robust ethical frameworks to guide the development and implementation of AI-CDSS in healthcare resource allocation. These frameworks should address issues of fairness, transparency, accountability, and patient-centeredness. While Char et al.‘s [6] work on ethical challenges in implementing machine learning in healthcare provides a valuable starting point, our findings suggest several specific additions to build a more comprehensive framework. Based on our participants’ experiences, such a framework should include: (1) explicit protocols for reviewing AI recommendations in cases involving vulnerable populations, as demonstrated by our participants’ practices in resource-constrained settings; (2) clear guidelines for documenting and justifying override decisions, as illustrated by clinicians’ experiences with maintaining professional autonomy; (3) structured processes for regular monitoring of AI recommendations’ impact on different patient populations, following the informal tracking systems developed by our participants; and (4) specific provisions for maintaining flexibility in resource allocation decisions to accommodate individual patient circumstances, as evidenced by our participants’ experiences in balancing cost-effectiveness with patient-specific needs. These additions would help translate theoretical ethical principles into practical implementation guidelines.

Second, our results highlight the importance of ongoing education and training for healthcare professionals on the ethical implications of AI in healthcare. This aligns with recommendations from Kolachalama and Garg [15] for integrating AI education into medical curricula. Based on our participants’ experiences, comprehensive AI literacy programs should focus on several key areas: (1) understanding how to evaluate and explain AI recommendations in resource allocation decisions, as highlighted by clinicians who struggled with communicating AI-assisted decisions to patients; (2) developing skills for identifying potential biases in AI recommendations, following the practices of participants who created informal monitoring systems; (3) building competency in determining when to override AI recommendations based on patient-specific factors, as demonstrated by practitioners who developed their own protocols for maintaining clinical judgment; and (4) understanding the relationship between data inputs and resource allocation decisions, addressing the challenges participants faced in explaining these connections to patients. Several institutions in our study had already begun implementing such focused training initiatives, with one administrator (Participant 7) describing workshops specifically designed to help staff understand the algorithms and engage critically with AI recommendations.

Third, our findings emphasize the need for interdisciplinary collaboration in the development and implementation of AI-CDSS. While Yu et al. [31] provide a foundation for promoting interdisciplinary collaboration in AI healthcare research, our findings suggest several specific collaborative structures needed in practice. These include: (1) regular forums for clinicians and technical teams to review and refine AI recommendations, as evidenced by participants who developed informal review processes; (2) structured collaboration between ethics committees and clinical teams in developing override protocols, reflecting the experiences of participants who created their own decision-making frameworks; (3) partnerships between privacy officers and clinical teams to address data sharing concerns, as highlighted by participants struggling with explaining data usage to patients; and (4) joint working groups of administrators and frontline providers to develop context-appropriate implementation strategies, following the example of institutions in our study that implemented regular team discussions about AI recommendations. As one hospital administrator (Participant 7) demonstrated through their institution’s workshop approach, successful implementation requires ongoing dialogue between technical experts who understand the algorithms and healthcare professionals who understand patient care contexts.

Finally, our results underline the importance of ongoing monitoring and evaluation of AI-CDSS to detect and mitigate unintended consequences. While Gianfrancesco et al. [11] provide a general framework for evaluating AI implementation, our findings highlight several specific evaluation priorities: (1) systematic tracking of AI recommendations’ impacts on vulnerable populations, following the practice of participants who developed informal monitoring systems to identify potential disparities; (2) documentation and analysis of override decisions, as illustrated by the department head (Participant 11) who tracked cases where AI recommendations misaligned with patient needs; (3) regular assessment of how AI recommendations affect resource distribution across different healthcare settings, reflecting the concerns raised by participants in resource-constrained environments; and (4) monitoring of patient responses to AI-influenced resource allocation decisions, addressing the challenges described by participants in maintaining patient trust. These evaluation components were exemplified by participants who implemented various monitoring approaches, from monthly audit meetings to detailed tracking systems for AI-assisted decisions.

We believe that our study underscores the importance of acknowledging the deductive nature of research designs that use structured interview protocols. While these designs ensure comprehensive coverage of known ethical challenges, they may constrain participants’ ability to introduce novel issues or challenge pre-existing assumptions. This necessitates careful framing of findings to reflect their rootedness in predefined themes. Additionally, the reflections shared by participants highlight the critical need for ongoing training and education. For instance, Participant 20 proposed the integration of ‘AI literacy’ programs into medical training to empower clinicians to critically evaluate AI recommendations. This aligns with the work of Kolachalama and Garg [15], who emphasize the need for a robust understanding of AI’s strengths and limitations among healthcare providers. Finally, participants’ emphasis on equity, transparency, and accountability calls for institutional frameworks that not only guide AI-CDSS implementation but also foster an environment of ethical reflection and shared decision-making. Drawing from Rudin’s [27] recommendation to prioritize interpretable AI models, future efforts should focus on designing systems that healthcare professionals can critically engage with, ensuring that these technologies enhance rather than undermine professional autonomy.

A critical question in empirical ethics is how descriptive findings can inform normative frameworks without falling into the is-ought gap. In this study, we describe how healthcare professionals perceive the ethical challenges of integrating AI-CDSS into resource allocation. These descriptive findings highlight specific areas of concern, such as equity, transparency, and shifting roles, which could serve as focal points for developing ethical frameworks. However, we recognize that empirical findings alone cannot determine normative principles. To bridge this gap, future work should adopt an interdisciplinary approach, integrating these stakeholder perspectives with established ethical theories such as Beauchamp and Childress’ principles of biomedical ethics [3] or Rawlsian theories of justice. For example, participants’ concerns about equity could inform the operationalization of distributive justice principles in the context of AI. Similarly, their emphasis on transparency aligns with the ethical imperative for explicability in AI systems, as highlighted in the literature [14]. While this study does not claim to develop a prescriptive ethical framework, it identifies key areas where empirical insights can inform theoretical work and policy development. Moving forward, stakeholder perspectives must be synthesized with normative theories, pilot-tested in real-world settings, and iteratively refined to ensure that ethical frameworks are both theoretically robust and practically applicable.

## Conclusion

The integration of AI-driven Clinical Decision Support Systems (AI-CDSS) into healthcare resource allocation presents both significant opportunities and complex ethical challenges. Our study has illuminated the multiple perspectives of healthcare professionals on this critical issue, revealing a better understanding of the potential benefits and risks associated with these technologies.

The key themes that emerged from our analysis - balancing efficiency and equity, ensuring transparency and explicability, navigating shifting roles and responsibilities, addressing ethical considerations in data usage and algorithm development, and maintaining patient-centered care in the face of cost-effectiveness pressures - underscore the need for a thoughtful and multidisciplinary approach to the implementation of AI-CDSS in healthcare.

As we move forward, it is crucial that we develop robust ethical frameworks, enhance AI literacy among healthcare professionals, foster interdisciplinary collaboration, and implement rigorous monitoring and evaluation processes. These steps will be essential in harnessing the potential of AI-CDSS to improve healthcare efficiency and outcomes while safeguarding against unintended consequences and ethical pitfalls.

Ultimately, the successful integration of AI-CDSS into healthcare resource allocation will require ongoing dialogue, careful consideration of ethical implications, and a commitment to preserving the fundamental values of equity, transparency, and patient-centered care that are at the heart of healthcare delivery. By addressing these challenges proactively, we can work towards a future where AI enhances rather than diminishes the quality and ethical standards of healthcare.

## Supplementary Information

Supplementary Material 1.

Supplementary Material 2.

## Data Availability

Survey data is available upon request from the corresponding author.
