# Supplementary material for: Ethical implications of AI-driven clinical decision support systems on healthcare resource allocation: a qualitative study of healthcare professionals’ perspectives
Source: BMC Med Ethics. 2024 Dec 21;25:148. doi: 10.1186/s12910-024-01151-8 (PMC11662436; doi:10.1186/s12910-024-01151-8)
Supplement: Supplementary file 1 — Supplementary Material 1. [file 12910_2024_1151_MOESM1_ESM.docx]

**Survey: Ethical Implications of AI-CDSS in Healthcare Resource Allocation**

**Introduction:** Thank you for participating in this study. The purpose of this interview is to gather your insights on the ethical implications of AI-driven Clinical Decision Support Systems (AI-CDSS) in healthcare resource allocation. Your responses will remain confidential and contribute to understanding how these systems impact ethical decision-making in healthcare.

**Section 1: General Background**

1. What is your current role in healthcare? (e.g., physician, nurse, healthcare administrator, medical ethicist)
2. How many years of experience do you have in your field? What is your age?
3. Have you had any direct experience with AI-CDSS in your practice? If yes, please describe.

**Section 2: Perceptions of AI-CDSS** 4. What are your general thoughts on the use of AI in healthcare decision-making? 5. In your experience, what are the primary benefits and challenges of implementing AI-CDSS in healthcare?

**Section 3: AI-CDSS and Resource Allocation** 6. How do you feel about the use of AI-CDSS for healthcare resource allocation (e.g., determining which patients receive specific treatments or resources)? 7. Do you believe AI-CDSS could exacerbate or mitigate existing healthcare disparities? Please elaborate. 8. What concerns do you have regarding the fairness and equity of AI-CDSS in resource allocation decisions?

**Section 4: Ethical Considerations** 9. How important is transparency and explicability in AI-CDSS? Why? 10. What are your thoughts on the privacy and consent implications of using patient data in AI-CDSS? 11. In your opinion, how should AI-CDSS balance cost-effectiveness with patient-centered care?

**Section 5: Professional Roles and Responsibilities** 12. How do you think AI-CDSS will impact clinical decision-making processes in the future? 13. Do you foresee any changes in the roles and responsibilities of healthcare professionals due to AI-CDSS? If so, how? 14. Who do you believe should be held accountable if an AI-CDSS contributes to a negative outcome?

**Section 6: Recommendations for Ethical Implementation** 15. What ethical framework would you recommend for the integration of AI-CDSS into healthcare resource allocation? 16. What steps do you believe healthcare institutions should take to ensure AI-CDSS is used responsibly?
