# Supplementary material for: Ethical implications of AI-driven clinical decision support systems on healthcare resource allocation: a qualitative study of healthcare professionals’ perspectives
Source: BMC Med Ethics. 2024 Dec 21;25:148. doi: 10.1186/s12910-024-01151-8 (PMC11662436; doi:10.1186/s12910-024-01151-8)
Supplement: Supplementary file 2 — Supplementary Material 2. [file 12910_2024_1151_MOESM2_ESM.docx]

**Appendix**

This comprehensive appendix provides additional context and detail to support the main findings of the study, offering readers a deeper understanding of the research process, participant characteristics, and key concepts related to the ethical implications of AI-CDSS in healthcare resource allocation.

Appendix A: Interview Guide

1. Introduction and Warm-up
   - Brief explanation of the study purpose
   - Participant's background and experience with AI-CDSS
2. General Perceptions of AI-CDSS in Healthcare
   - Overall thoughts on the role of AI in healthcare decision-making
   - Perceived benefits and challenges of AI-CDSS implementation
3. AI-CDSS and Resource Allocation
   - Views on using AI-CDSS for healthcare resource allocation decisions
   - Potential impacts on healthcare equity and access
4. Ethical Considerations
   - Transparency and explicability of AI-CDSS decision-making processes
   - Data privacy and consent issues in AI-CDSS development and use
   - Balancing cost-effectiveness and patient-centered care
5. Professional Roles and Responsibilities
   - Impact of AI-CDSS on clinical decision-making processes
   - Changes in professional roles and responsibilities with AI integration
   - Accountability and liability concerns
6. Training and Competency
   - Necessary skills and knowledge for working with AI-CDSS
   - Views on AI education in healthcare professional training
7. Future Directions
   - Anticipated developments in AI-CDSS for resource allocation
   - Recommendations for ethical implementation of AI-CDSS
8. Closing
   - Additional thoughts or concerns
   - Thank you and next steps

Appendix B: Thematic Analysis Process

1. Data Familiarization
   - Transcription of interviews
   - Multiple readings of transcripts by authors.
2. Initial Coding
   - Development of initial codebook
   - Independent coding of transcripts by two researchers
   - Regular meetings to discuss and refine codes
3. Theme Development
   - Grouping of related codes into potential themes
   - Creation of thematic maps to visualize relationships
4. Theme Refinement
   - Review of themes in relation to coded extracts and entire dataset
   - Refinement of theme definitions and names
5. Final Analysis
   - Selection of illustrative quotes for each theme
   - Writing of detailed analysis for each theme

Appendix C: Coding Framework

Table C1: Final Coding Framework

| **Theme** | **Subthemes** | **Example Codes** |
| --- | --- | --- |
| Efficiency vs. Equity | - Potential for optimized resource allocation<br>- Concerns about exacerbating disparities | - AI efficiency<br>- Healthcare equity<br>- Resource optimization<br>- Disparity concerns |
| Transparency and Explicability | - Need for interpretable AI models<br>- Challenges of "black box" algorithms | - AI transparency<br>- Explainable AI<br>- Algorithm opacity<br>- Decision justification |
| Shifting Roles and Responsibilities | - Changes in clinical decision-making<br>- Questions of accountability | - Professional autonomy<br>- AI-human collaboration<br>- Liability concerns<br>- Role redefinition |
| Data Ethics | - Patient privacy and consent<br>- Data representation and bias | - Data privacy<br>- Informed consent<br>- Algorithmic bias<br>- Data diversity |
| Patient-Centered Care | - Balancing cost-effectiveness and individualized care<br>- Maintaining empathy in AI-assisted care | - Cost-effectiveness<br>- Personalized medicine<br>- Empathetic care<br>- Patient preferences |

Appendix D: Illustrative Quotes

Table D1: Additional Illustrative Quotes by Theme

| **Theme** | **Quote** | **Participant** |
| --- | --- | --- |
| Efficiency vs. Equity | "AI could help us do more with less, but we need to be vigilant about who benefits and who might be left behind." | Participant 13, Hospital Administrator |
| Transparency and Explicability | "If I can't explain to a patient how we arrived at a decision, I'm not comfortable using that system, no matter how accurate it claims to be." | Participant 6, Primary Care Physician |
| Shifting Roles and Responsibilities | "We're not just implementing a new technology; we're potentially redefining what it means to be a healthcare provider." | Participant 19, Medical Ethicist |
| Data Ethics | "The quality of our AI is only as good as the data we feed it. We have a ethical obligation to ensure that data is representative and ethically sourced." | Participant 21, Healthcare Data Scientist |
| Patient-Centered Care | "The challenge is to use AI to enhance, not replace, the human touch in medicine. We can't lose sight of the individual in pursuit of population-level efficiencies." | Participant 4, Oncology Nurse |

Table E1: Examples of Original vs. Edited Quotes

Original quote: AI systems, well, they process a lot of data, and they’re supposed to help us see where resources might be underused or overused. That’s helpful, I guess, but sometimes I wonder—are we just moving resources around without really fixing the bigger problem of fairness? It’s hard to say if it’s actually solving the equity issue or just making things look more efficient on paper." (Participant 7, Hospital Administrator)

Edited Quote: AI systems can process vast amounts of data to identify areas where resources are being underutilized or overutilized, potentially leading to more efficient allocation.

Original Quote: When I’m using an AI system, I need to be able to explain its recommendations—to my patients, to my team—but sometimes I can’t. I mean, the system might say, ‘This patient should be prioritized,’ but it doesn’t really tell me why. Is it their condition? Their age? Something else? And if I can’t explain it, then how can I justify the decision to others?" (Participant 12, Neurologist)

Edited Quote: If I'm going to rely on an AI system to help me make decisions about resource allocation, I need to be able to understand and explain its reasoning to my patients and colleagues. (Participant 12, Neurologist)

Original Quote: These systems are useful, sure—they give you insights you might not think of on your own—but at the end of the day, it’s still my job to make the decision. And that’s hard because I have to balance what the AI says with what I know about the patient, their context, and, well, just being empathetic. It’s tricky—sometimes it feels like the AI and my clinical judgment are in two different worlds." (Participant 15, Critical Care Nurse)

Edited Quote: While these systems can provide valuable insights, we can't lose sight of the importance of human empathy and contextual understanding in healthcare decisions." (Participant 15, Critical Care Nurse)
